# Adult psychological outcomes among women with different adolescent PCOS presentations

**DOI:** 10.3389/fendo.2026.1793628

**Published:** 2026-09-04

**Authors:** Maria Małgorzata Nowak-Ciołek, Julia Paczyna, Katarzyna Grygiel, Julia Sokal, Żaneta Malczyk, Karolina Skrzyńska, Agnieszka Zachurzok

**Affiliations:** 1 Students' Scientific Association at the Department of Pediatrics, Faculty of Medical Sciences in Zabrze, Medical University of Silesia in Katowice, Zabrze, Poland; 2 Department of Pediatrics, Faculty of Medical Sciences in Zabrze, Medical University of Silesia in Katowice, Zabrze, Poland; 3 Department of Pediatric and Pediatric Endocrinology, Faculty of Medical Sciences in Katowice, Medical University of Silesia, Katowice, Poland

**Keywords:** adolescence, depressive symptoms, mental health, PCOS, quality of life

## Abstract

**Introduction:**

Polycystic ovary syndrome (PCOS) is a prevalent endocrine disorder, often beginning in adolescence and associated with reproductive, metabolic, and psychological complications. The present study evaluated whether adolescent patients meeting the 2023 adolescent-specific PCOS criteria (Rotterdam phenotypes A and B) exhibit poorer mental health or lower quality of life (QoL) in adulthood compared with those with Rotterdam PCOS phenotypes C and D.

**Methods:**

A retrospective analysis was conducted among women previously hospitalized in pediatric endocrinology wards and diagnosed with PCOS during adolescence. Clinical data, hormonal profiles, and comorbidities were extracted from medical records. The study group [SG] comprised patients with Rotterdam PCOS phenotypes A and B, while the comparison group [CG] included phenotypes C and D and adolescents at risk of PCOS. In adulthood, participants completed a 66-item survey including self-reported BMI, menstrual symptoms, Ferriman-Gallwey scoring, comorbidity assessment, the Patient Health Questionnaire-9 (PHQ-9), and the WHOQOL-BREF.

**Results:**

A total of 46 individuals completed the survey, including 32 participants in the SG and 14 in the CG. The median age was 22.5 years in the SG and 21.5 years in the CG. All participants were Polish. During adolescence, SG and CG differed in testosterone levels (p = 0.0007) and number of patients with menstrual irregularities (p = 0.006). In adulthood, no significant between-group differences were observed in BMI, Ferriman-Gallwey scores, PHQ-9 outcomes, or WHOQOL-BREF domain scores. The distribution of depressive symptom severity did not differ significantly. Moreover, no correlations were found between QoL or depressive symptoms and BMI change, disease duration or hirsutism. However, mean PHQ-9 scores in both groups indicated clinically relevant depressive symptoms.

**Conclusions:**

Adult women diagnosed with PCOS (Rotterdam phenotypes A and B) in adolescence did not exhibit poorer quality of life or increased depressive symptoms compared with women presenting Rotterdam PCOS phenotypes C and D or adolescents previously classified as being at risk of PCOS. No significant differences were observed between groups in PHQ-9 or WHOQOL-BREF scores, nor were these outcomes related to BMI, hyperandrogenism, or disease duration. Both groups demonstrated elevated depressive symptom levels, highlighting the need for longitudinal studies on long-term psychological outcomes in PCOS.

## 1 Introduction

Polycystic ovary syndrome (PCOS) is one of the most common endocrine disorders in women, linked to an increased risks for obesity or overweight, glucose intolerance, type 2 diabetes, dyslipidemia, and metabolic-associated steatotic liver disease (1).

Research on mental health outcomes in PCOS patients has yielded inconsistent findings, partly due to varying data between adolescent and adult populations (2, 3). However, a meta-analysis by Brutocao indicated that adult women with PCOS show a higher prevalence of psychiatric diagnoses, including depression, anxiety, bipolar disorder, and obsessive-compulsive disorder, compared with healthy controls (4). In addition, several studies have demonstrated that adult women with PCOS experience a significantly lower quality of life (QoL), particularly in relation to physical health, body image, and emotional well-being (5–7).

Ibanez et al. and, subsequently, Teede et al. were the first to emphasize that the symptoms of PCOS overlap with the mild hormonal and clinical variations observed during adolescence and therefore proposed different diagnostic criteria for PCOS in adolescent girls (8, 9). In 2023, updated diagnostic criteria for PCOS in adolescents were introduced, specifying that both menstrual irregularity and clinical or biochemical hyperandrogenism are necessary for diagnosis, while polycystic ovarian morphology is excluded as a criterion (10). According to these revised criteria, a diagnosis of PCOS in adolescents is made only when the diagnostic features correspond to Rotterdam phenotype A or phenotype B. Such diagnostic criteria minimize the risk of over-diagnosing PCOS in adolescents and allow for the identification of those at increased future health risk. This is consistent with findings by Tay et al., where BMI trajectories in women who did not meet the adolescent diagnostic criteria were comparable to controls, whereas those who did meet the criteria had significantly higher BMI in adulthood (11).

The objective of this study is to investigate whether adolescent patients meeting the diagnostic criteria for PCOS (Rotterdam phenotypes A and B) have an increased risk of developing psychiatric symptoms and poor quality of life as adults compared to adolescents meeting Rotterdam PCOS criteria for phenotypes C and D or those classified as being at risk of PCOS.

## 2 Materials and methods

A retrospective analysis of the medical records of adolescent patients with PCOS was performed. Patients were identified through the hospital database (2011 - 2019) and had been diagnosed with PCOS during that period according to the Rotterdam criteria. We extracted data on: age, weight, height, body mass index (BMI), regularity of menstrual cycle, pelvis ultrasound findings, assessment using the Ferriman-Gallwey scale, comorbidities, blood test results: testosterone, 17 hydroxyprogesterone (17OHP), dehydroepiandrosterone sulfate (DHEAS), androstenedione concentrations.

Exclusion criteria included: eating disorders, Cushing’s syndrome diagnosis, adrenal cortex dysfunction (defined as 17-OHP concentration above 2 ng/mL in the early follicular phase), diagnosis of adrenal or ovarian neoplasms, uncontrolled thyroid dysfunction (hypothyroidism or hyperthyroidism), hyperprolactinemia (concentration of prolactin (PRL) ≥ 36 µg/L) and pregnancy.

All of the patients in study group (SG) fulfilled the updated diagnostic criteria for adolescents outlined below:

1. Gynecological age above 2 years;

2. Specific criteria for menstrual disorders:

• 1 year and <3 years post menarche with menstrual cycle lengths < 21 days or >45 days

• 3 years post menarche with menstrual cycle lengths < 21 days and > 35 days OR fewer than 8 menstrual cycles per year

• more than 90 days of menstrual cycle in 1 year

• amenorrhea 3 years after thelarche

2. Hyperandrogenism:

a. biochemical: elevated levels of testosterone or 17OHP, androstenedione, DHEAS

b. clinical: hirsutism (Ferriman-Gallwey Score ≥ 8 points)

The comparison group (CG) comprised adolescent girls showing clinical features indicative of PCOS but not meeting the full diagnostic criteria for adolescent girls, and who did not present with any conditions defined in the exclusion criteria (phenotypes C, D according to Rotterdam criteria or only one feature of PCOS).

### 2.1 Assessment

Participants completed a 66-question survey divided into six sections:

1. Consent - Agreement to participate in the study.
2. Self-measurements - Participants recorded their body weight and height according to the provided instructions. Based on the data provided, BMI was calculated.
3. PCOS Symptom Self-Assessment - Including hirsutism evaluation via the pictorial Ferriman-Gallwey scale and assessment of menstrual cycle regularity.
4. Comorbidity Prevalence - Assessment of additional health conditions co-occurring with PCOS, including infertility-related factors such as reproductive intentions, history of pregnancies, and pregnancies carried to term.
5. World Health Organization Quality of Life BREF Questionnaire - This standardized self-report tool consists of 26 items covering four major domains: physical health, psychological well-being, social relationships, and environmental factors. Each item is rated on a 5-point Likert scale, reflecting the respondent’s perception of their quality of life over the preceding two weeks. Raw domain scores were calculated according to the WHOQOL-BREF scoring guidelines and transformed into a 0–100 scale using the validated WHO conversion table, where higher scores indicate better perceived quality of life (12–16).
6. Patient Health Questionnaire-9 (PHQ-9) - It consists of nine items corresponding to the diagnostic criteria for major depressive disorder outlined in the Diagnostic and Statistical Manual of Mental Disorders (DSM-IV). Each item is rated on a 4-point Likert scale reflecting symptom frequency over the previous two weeks (0 = “not at all,” 1 = “several days,” 2 = “more than half the days,” and 3 = “nearly every day”). The total score ranges from 0 to 27, with higher scores indicating greater severity of depressive symptoms. Score: <5 points without depression, 5–9 is mild depression; 10–14 is moderate depression; ≥15 is considered severe depression (6, 17–22).

The questionnaire was anonymous and not publicly accessible; each participant received an individual access token to ensure controlled participation and prevent multiple entries. There was no time limit for survey completion. The estimated completion time was approximately 15 minutes. Participants were able to pause and resume the survey at any point to ensure convenience and completeness of responses.

Statistical analysis was conducted using Statistica 13.3 PL software. The SG and CG were compared using either the Mann-Whitney U-Test or the Student’s t-test for independent samples, depending on data distribution as assessed by the Shapiro-Wilk test. For the correlation analyses, we used the Pearson correlation, and the Spearman Rank correlation based on the data distribution. The Fisher’s exact test was used for the analysis of categorical variables with small sample sizes or expected cell frequencies below five. Bonferroni correction was not applied because this study was exploratory, involved a small sample size, and aimed primarily to detect potential psychological risk signals that warrant validation in larger cohorts.

## 3 Results

A total of 112 patients meeting the inclusion criteria were identified from two pediatric endocrinology wards. Of these, 42 did not respond to any contact attempts, 22 declined participation (12 met the criteria for the comparison group and 10 for the study group), and two failed to complete the survey, resulting in a final sample of 46 patients included in the analysis. Most patients who declined participation chose not to disclose the reason for their decision.

The study group consisted of 32 young women aged between 18 and 26 years (median: 22.5). Of the 32 participants, 5 met the criteria for Rotterdam phenotype A, while remaining 27 were classified as phenotype B. The comparison group consisted of 14 young women, aged between 20 and 26 years (median: 21.5). In this group, during adolescence, 2 girls met the criteria for Rotterdam phenotype C, and 8 met the criteria for phenotype D. The remaining 4 adolescents were considered as “at risk of PCOS”, because of symptoms and exclusion of other illnesses (**Table 1**).

**Table 1:** Symptoms and associated phenotypes in patients.

Group	Symptoms/Rotterdam’s phenotype	HA	AO	PCOM	Number of adolescents patients
SG	A	**+**	**+**	+	5/46
	B	**+**	**+**		27/46
CG	C	+		+	2/46
	D		+	+	8/46
	Not classified		+		2/46
	Not classified	+			2/46
	Summary	36/46	42/46	15/46	

Patients overlapping Rotterdam and new criteria are highlighted in blue. SG, study group; CG, comparison group; HA, hyperandrogenism; AO, oligo-anovulation; PCOM, polycystic ovary morphology.

Among adolescents, the significant between-group differences were observed in testosterone levels and number of patients with menstrual irregularities (**Table 2**).

**Table 2:** General characteristics and comparison between the SG and the CG during adolescence.

Patients characteristics	SG n=32	CG n=14	p value
Age (years)	16.0 [15.4 - 16.7]	16.5 [15.5 - 17.0]	0.33
BMI (kg/m2)	27.3 ± 7.0	26.0 ± 5.1	0.55
Testosterone (ng/dl)	67.8 ± 23.9	37.4 ± 16.9	**0.0007**
Ferriman-Gallwey score	9.0 [7.0 - 13.0]	5.0 [5.0 - 12.0]	0.23
Number of patients with menstrual irregularities	32	10	**0.006#**

Group differences were analyzed using the Student’s t-test or the Mann-Whitney U test, depending on data distribution. Results analyzed with the Student’s t-test are presented as mean ± standard deviation (SD), whereas results analyzed with the Mann-Whitney U test are expressed as median values with interquartile ranges; # - Fisher exact test. Bold values indicate statistically significant differences.

Comorbidities were assessed based on patient self-report. In the SG, 14 (43.8%) participants reported additional health conditions (8 with insulin resistance, 4 with impaired fasting glucose, and 4 with lipid abnormalities). In the CG, 7 (50%) participants reported comorbidities (4 with insulin resistance, 2 with impaired fasting glucose, and 2 with lipid abnormalities). Medication history revealed that 8 patients were treated with metformin (6 in the SG and 2 in the CG), and 1 patient in the SG received semaglutide. In addition, 10 participants confirmed having psychiatric disorders (8 in the SG and 2 in the CG), of whom 3 were undergoing antidepressant therapy (2 in the SG and 1 in the CG). Infertility was reported by 2 participants, denied by 6, and deemed not applicable by the remaining members of the cohort.

In our cohort, menstrual irregularities decreased from 32 in adolescence to 11 in adulthood in the SG, and from 10 to 4 in the CG. However, this change cannot be reliably interpreted, as our dataset contains information only on contraceptive therapy (used by 12 patients in the SG and 8 in the CG) and lacks data on other factors that may influence menstrual regularity.

Statistical analysis comparing the study and comparison groups did not reveal any significant differences between groups (including age, BMI and Ferriman-Gallwey scale scores) (**Table 3**).

**Table 3:** General characteristics and comparison between the SG and the CG during adulthood. Group differences were analyzed using the Student’s t-test or the Mann-Whitney U test, depending on data distribution.

Patients characteristics	SG n=32	CG n=14	p value
Age (years)	22.5 [20.5 - 24.0]	21.5 [21.0 - 23.0]	0.75
Duration of illness (years)	6.0 [5.0 - 8.0]	5.5 [5.0 - 6.5]	0.63
BMI (kg/m2)	26.0 [21.5 - 34.8]	24.0 [22.0 - 30.0]	0.53
Δ BMI	1.9 ± 4.1	0.3 ± 4.2	0.23
Ferriman-Gallwey score	16.6 ± 10.8	15.2 ± 10.2	0.67
Number of patients with menstrual irregularities	11	4	0.80#

Results analyzed with the Student’s t-test are presented as mean ± standard deviation (SD), whereas results analyzed with the Mann-Whitney U test are expressed as median values with interquartile ranges; # - Fisher exact test.

Health-related quality of life and depressive symptom severity, as assessed using the WHOQOL-BREF and PHQ-9 questionnaires, were compared between the SG and the CG. No statistically significant between-group differences were observed in mean WHOQOL-BREF scores. Similarly, PHQ-9 total scores were comparable between the SG and CG (**Table 4**).

**Table 4:** Survey results were compared between the SG and the CG during adulthood.

Questionnaires	SG n=32	CG n=14	p value
PHQ-9 summary	11.4 ± 6.4	11.64 ± 6.4	0.92
WHO QOL-BREF summary	54.3 ± 9.9	56.2 ± 11.0	0.55
Physical health (DOMAIN 1)	14.0 [11.5 - 16.0]	15.0 [13.0 - 15.0]	0.70
Psychological (DOMAIN 2)	13.0 [11.0 - 15.0]	14.0 [11.0 - 16.0]	0.46
Social relationships (DOMAIN 3)	15.0 [12.0 - 16.0]	15.0 [11.0 - 17.0]	0.77
Environment (DOMAIN 4)	14.0 [12.0 - 16.0]	15.0 [13.0 - 16.0]	0.71

Group differences were analyzed using the Student’s t-test or the Mann-Whitney U test, depending on data distribution. Results analyzed with the Student’s t-test are presented as mean ± standard deviation (SD), whereas results analyzed with the Mann-Whitney U test are expressed as median values with interquartile ranges.

Based on the known determinants of poor quality of life in PCOS, we analyzed the correlations between illness duration, BMI increase, Ferriman-Gallwey scores and questionnaire outcomes, including domain-specific WHOQOL-BREF scores. No statistically significant correlations were identified.

The Fisher’s exact test was used to compare the study and comparison groups across the different depression severity categories defined by PHQ-9 thresholds (<5 = no depression, 5-9 = mild, 10-14 = moderate, ≥15 = severe). No significant differences were observed.

In addition, an analysis was conducted using commonly applied cut-off values of ≥10 points for the PHQ-9 and ≤60 points for the WHOQOL-BREF, as reported in previous studies of PCOS populations. Using these thresholds, clinically relevant depressive symptoms were identified in 16 of 32 (50.0%) participants in the study group and 9 of 14 (64.3%) participants in the comparison group. Reduced quality of life, according to the WHOQOL-BREF cut-off, was observed in 11 of 32 (35.4%) participants in the study group and 5 of 14 (35.7%) participants in the comparison group. No statistically significant between-group differences were found in the prevalence of depressive symptoms (Fisher’s exact test, p = 0.52) or reduced quality of life (Fisher’s exact test, p = 0.59).

## 4 Discussion

In this study, we aimed to evaluate whether a diagnosis of PCOS based on adolescent-specific criteria (corresponding to Rotterdam phenotypes A and B) is associated with an increased risk of depressive symptoms and impaired quality of life in adulthood compared with women presenting subthreshold PCOS manifestations, including Rotterdam phenotypes C and D and adolescents previously classified as being at risk of PCOS. Adolescents meeting the updated diagnostic criteria for PCOS are considered to be at the highest risk of adverse long-term health outcomes, as demonstrated by Tay et al. and may therefore be theoretically predisposed to a greater psychological burden (11).

A retrospective analysis identified 46 women with varying severity of PCOS-related symptoms during adolescence: 32 met the adolescent-specific diagnostic criteria for PCOS (Rotterdam phenotypes A and B), whereas 14 did not meet these criteria (Rotterdam phenotypes C and D or adolescents at risk of PCOS). Patients completed the PHQ-9 and WHOQOL BREF questionnaires. No differences in adult quality of life or in the prevalence of depressive symptoms were observed between the study and comparison groups. Furthermore, no significant correlations were identified between quality of life, depressive symptoms, and their potential determinants.

Importantly, the present study did not include a healthy control group. Therefore, the findings should be interpreted as comparisons between women meeting adolescent-specific PCOS criteria and women with subthreshold PCOS-related clinical presentations, rather than comparisons with the general population.

### 4.1 Quality of life in PCOS patients

The WHOQOL questionnaire has been validated as a reliable instrument for assessing quality of life in women with PCOS. However, a standardized cut-off score for the classification of QoL levels has not yet been established. Silva et al. proposed a threshold of 60 points as optimal for adults, whereas Ishrat et al. defined scores < 50 as indicative of poor QoL among infertile women (11, 12). In contrast, several other investigations in PCOS cohorts interpreted QoL dimensionally, without categorical thresholds, by comparing mean domain values and assuming higher scores to reflect better perceived QoL (7, 14, 15).

In the present study, the abbreviated WHOQOL-BREF questionnaire was applied, and the scores were converted to WHOQOL equivalents using the validated WHO conversion table. No statistically significant differences were observed in either mean values or the proportion of participants scoring below 60 points.

Dilbaz et al. reported significantly poorer QoL in patients presenting with hyperandrogenism and anovulation, with BMI and hirsutism showing strong negative correlations with QoL indices (14). In our study, these relationships were not replicated. It should be noted, however, that studies by Ishrat et al. and Dilbaz et al. focused exclusively on infertile patients, in whom infertility constitutes a major determinant of diminished QoL (5, 13, 14). In our cohort, only two participants reported infertility, six reported no history of infertility, and for the remaining participants this issue was not applicable. Given the relatively young age of both the study and comparison groups, infertility was unlikely to have substantially influenced the observed WHOQOL-BREF outcomes.

Rzońca et al. indicated that both higher BMI and longer disease duration are associated with reduced life satisfaction in PCOS patients (16). Our findings did not replicate those results, which may be attributable to the absence of significant differences in ΔBMI between the SG and CG and shorter disease duration.

### 4.2 Depressive symptoms in PCOS patients

The Patient Health Questionnaire-9 (PHQ-9) remains one of the most frequently employed tools for evaluating depressive symptomatology in women with PCOS (6, 17–22). Most investigations apply a cut-off of ≥10 points to identify clinically relevant depressive symptoms. Bhattacharaya et al., in a study including healthy controls, reported a markedly increased risk of depression in women with PCOS, with no significant associations with age, marital status, education, or employment. Dayo et al. further demonstrated that distinct demographic and physiological characteristics among PCOS patients with depression correspond to specific depressive phenotypes (18, 19). In the current analysis, no statistically significant intergroup differences were detected in PHQ-9 total scores or in the distribution of depression-severity categories. Patients in our cohort exhibited mean PHQ-9 scores above the cut-off threshold for depression (SG = 11.4 ± 6.4, CG = 11.64 ± 6.4). Clinically relevant depressive symptoms were present in 50% of the SG and 64% of the CG. These findings indicate that elevated depressive symptom burden may be present in both women meeting adolescent-specific PCOS diagnostic criteria and those with subthreshold PCOS manifestations. Moreover, 10 participants (8 in the SG and 2 in the CG) had a diagnosed psychiatric disorder, and 3 (2 in the SG and 1 in the CG) were receiving pharmacological treatment.

In contrast, Krstić et al. observed no significant differences in PHQ-9 or GAD-7 scores between groups and no correlations with BMI, waist circumference, or androgen concentrations (21). Such discrepancies may reflect differences in methodology, population characteristics, and time context, as well as sociocultural and environmental factors.

Bibi et al. also noted that obesity is an important factor contributing to the intensification of depressive symptoms, as women with PCOS and coexisting obesity exhibited higher levels of depression compared to their non-obese counterparts (17). Our findings demonstrated no significant correlations between PHQ-9 scores and BMI, hirsutism or disease duration.

### 4.3 Phenotype-related differences in QoL and depressive symptoms

The comparison group in our study comprised individuals with PCOS phenotypes C and D as well as adolescents at risk of PCOS. Importantly, the comparison group should not be interpreted as a healthy control population. Although these individuals did not meet the updated adolescent-specific diagnostic criteria for PCOS, many continued to exhibit clinical features associated with PCOS, including hyperandrogenic manifestations. Consequently, both groups may have experienced a similar degree of PCOS-related symptom burden, which could have attenuated potential differences in quality of life and depressive symptoms. Therefore, the present findings should be interpreted as comparisons between women with varying severity of PCOS-related clinical presentations rather than between affected and unaffected individuals.

Evidence from adult populations indicates that no consistent differences in depression or anxiety prevalence are observed across PCOS phenotypes, including between NIH and non-NIH presentations (23, 24). Similarly, studies using the Rotterdam classification have reported no significant inter-phenotypic differences in depressive symptoms, anxiety or global quality of life measures (25, 26).

Phenotype-related differences appear to be largely confined to specific symptom-related quality of life domains. Hyperandrogenic phenotypes (A and B) have been associated with poorer hirsutism-related QoL, phenotype D with greater acne-related burden, and phenotype C with more pronounced menstrual-related impairment, while overall psychological outcomes remain comparable across phenotypes (26). Lower mental health scores have been reported in NIH-type PCOS compared with non-NIH phenotypes and reduced physical health scores in women with PCOS overall compared with controls (24).Collectively, these findings suggest that phenotypic classification mainly affects specific QoL domains rather than global depression, anxiety or overall quality of life.

In the present study, analyses encompassed both total scores and domain-specific outcomes of the applied questionnaires in relation to hyperandrogenic features and BMI. However, no statistically significant correlations were identified at either the total-score or domain level. Consequently, our results did not replicate previously reported domain-specific associations, further supporting the notion that phenotypic classification may have limited impact on psychological outcomes in this population.

### 4.4 Current strategies for PCOS symptoms management

Previous studies have identified hyperandrogenism, excess adiposity, and infertility as key contributors to impaired quality of life and increased depressive symptoms in women with PCOS. Although advances in treatment may influence long-term outcomes, the present study did not collect sufficient treatment-related data to evaluate this possibility.

Combined oral contraceptive pills are considered first-line pharmacological therapy for the management of menstrual irregularities, acne, and hirsutism in patients with PCOS (27–29). In our cohort, 12 patients in the SG and 8 in the CG reported the use of contraceptive therapy, with 10 individuals (6 in the SG and 4 in the CG) reporting subjective improvement in PCOS-related symptoms.

Regarding metabolic management, 8 patients were treated with metformin (6 in the SG and 2 in the CG) for insulin resistance, and 1 patient in the SG received semaglutide. Metformin remains widely used in the management of PCOS-related insulin resistance and metabolic dysfunction, particularly in patients with impaired glucose tolerance or obesity (8).

Infertility-related outcomes could not be meaningfully evaluated in our cohort, as infertility was reported by only 2 participants, denied by 6, and considered not applicable by the remaining individuals.

## 5 Conclusions

In this study, a diagnosis of PCOS based on adolescent-specific criteria was not associated with higher PHQ-9 or lower WHOQOL-BREF scores compared with women with subthreshold PCOS manifestations in adulthood. No significant differences were observed between the study and comparison groups in PHQ-9 or WHOQOL-BREF outcomes, nor were these measures correlated with BMI, hyperandrogenic features, or disease duration. Participants in both groups demonstrated mean PHQ-9 scores within the range typically considered clinically relevant, despite the absence of significant differences between women meeting and not meeting adolescent PCOS diagnostic criteria. The lack of between-group differences may indicate that factors other than adolescent PCOS phenotype contribute to adult psychological outcomes; however, the present study was not designed to identify such determinants. Further longitudinal studies are warranted to clarify long-term psychological trajectories following adolescent PCOS diagnosis.

## 6 Limitations of the study

It must be acknowledged that our study included a comparison group rather than a fully healthy control group, as participants were not entirely free from medical or psychological conditions. This limits the ability to draw conclusions about differences between PCOS patients and completely healthy peers. It should be noted that unsuccessful contact attempts were largely attributable to the availability of caregivers’ telephone numbers only, as participants had been adolescents at earlier stages of the project. We highlight this issue to emphasize that longitudinal and follow-up studies in this population may face substantial challenges in participant inclusion. Future research should therefore consider recruiting larger initial cohorts to mitigate the impact of attrition and non-contact on study outcomes. Moreover, selection bias cannot be excluded. Individuals experiencing either greater symptom burden or greater health awareness may have been more likely to participate. Another limitation of this study is the reliance on self-assessment tools, including the Ferriman-Gallwey scale for hirsutism and self-reported questionnaires such as the PHQ-9 and WHOQOL-BREF. Although these instruments are validated and widely used in both clinical and research settings, self-administered assessments may introduce subjective bias and recall bias. Participants might under- or overestimate the severity of their symptoms due to differences in self-perception, cultural or aesthetic attitudes toward body image, limited understanding of medical terminology, or inaccurate recall of past symptoms and experiences. Additionally, the absence of clinician-confirmed scoring for the Ferriman-Gallwey scale could reduce objectivity in evaluating hyperandrogenic features. Such factors may contribute to variability in the data and should be considered when interpreting the results. No Bonferroni correction or other family-wise error rate adjustments for multiple comparisons were applied. The relatively small sample size, particularly within the comparison group, may have limited statistical power and increased the risk of Type II error. Consequently, potentially meaningful differences between groups may have remained undetected. The absence of statistically significant differences should not be interpreted as evidence of equivalence between groups. Accordingly, the findings should be interpreted with appropriate caution.

## Data Availability

The original contributions presented in the study are included in the article/supplementary material. Further inquiries can be directed to the corresponding author.
